# New dienelactone hydrolase from microalgae bacterial community-Antibiofilm activity against fish pathogens and potential applications for aquaculture

**DOI:** 10.1038/s41598-023-50734-9

**Published:** 2024-01-03

**Authors:** Lutgardis Bergmann, Simone Balzer Le, Gunhild Hageskal, Lena Preuss, Yuchen Han, Yekaterina Astafyeva, Simon Loevenich, Sarah Emmann, Pablo Perez-Garcia, Daniela Indenbirken, Elena Katzowitsch, Fritz Thümmler, Malik Alawi, Alexander Wentzel, Wolfgang R. Streit, Ines Krohn

**Affiliations:** 1 Department of Microbiology and Biotechnology, Institute of Plant Science and Microbiology, University of Hamburg, Ohnhorststr.18, 22609 Hamburg, Germany; 2 Department of Biotechnology and Nanomedicine, SINTEF Industry, Trondheim, Norway; 3 Molecular Microbiology, Institute for General Microbiology, Kiel University, Kiel, Germany; 4 Bioinformatics Core, University Medical Center Hamburg-Eppendorf, Hamburg, Germany; 5 Virus Genomics, Leibniz Institute of Virology, Hamburg, Germany; 6 Core Unit Systems Medicine, University of Würzburg, Würzburg, Germany

**Keywords:** Microbiology, Molecular biology

## Abstract

Biofilms are resistant to many traditional antibiotics, which has led to search for new antimicrobials from different and unique sources. To harness the potential of aquatic microbial resources, we analyzed the meta-omics datasets of microalgae-bacteria communities and mined them for potential antimicrobial and quorum quenching enzymes. One of the most interesting candidates (Dlh3), a dienelactone hydrolase, is a α/β-protein with predicted eight α-helices and eight β-sheets. When it was applied to one of the major fish pathogens, *Edwardsiella anguillarum*, the biofilm development was reproducibly inhibited by up to 54.5%. The transcriptome dataset in presence of Dlh3 showed an upregulation in functions related to self-defense like active genes for export mechanisms and transport systems. The most interesting point regarding the biotechnological potential for aquaculture applications of Dlh3 are clear evidence of biofilm inhibition and that health and division of a relevant fish cell model (CHSE-214) was not impaired by the enzyme.

## Introduction

Fish and other aquatic animals or plants are among others fundamental to feed the growing global population. Total fisheries and aquaculture production reached an all-time record of 214 million tons in 2020, comprising 178 million tons of aquatic animals and 36 million tons of algae^1^. At present, half of the worlds fish and aquatic animals supply derives from aquaculture, with land-based aquaculture growing even faster than marine aquaculture^1^. Intensifying fish farming comes with considerable challenges. Simple fish farm facilities, like open ponds, often develop high levels of organic matter over time as a consequence of feeding, fish growth, waste accumulation, and is threatened by increasing fish mortality due to diseases caused by microorganisms^2–4^.

Outbreaks of bacterial fish diseases are a major cause for mortality aquaculture^3^. Especially in land-based recirculating aquaculture systems (RAS), biofilms on tanks, pipes and in filtration systems can serve as reservoirs for bacterial pathogens that can be difficult to eradicate. Over the years, the number of bacterial species associated with fish disease has been steadily increasing^5^. For example, *Vibrio anguillarum* is recognized as an important pathogen in fish. It causes vibriosis or hemorrhagic septicemia in various aquatic species, including marine and freshwater fish, shellfish, and other aquatic organisms. In addition, *Tenacibaculum*, formerly known as *Flexibacter maritimus*, is responsible tenacibaculosis, a disease commonly known as marine flexibacteriosis or columnaris disease. It affects marine and freshwater fish and leads to skin ulcers, lesions, fin erosion, and gill damage. *Moritella* species are most problematic in cold water environments. *Moritella viscosa* in particular has been associated with causing winter ulcers in various fish species, especially salmonid fish^5^. In addition, one of the oldest described pathogens is *Aeromonas salmonicida,* the bacterium causing furunculosis in salmonids^6,7^. Other examples are *Flavobacterium columnare,* the bacterium responsible for columnaris disease in various wild and freshwater fish species such as carp, salmonids and eel^8^, *Flavobacterium psychrophilum* leading to bacterial cold-water disease (BCWD) and Rainbow trout fry syndrome (RTFS) in farmed rainbow trout, *Yersinia ruckeri*, causing serious septicemic bacterial disease of salmonid fish^9^, as well as *Pseudomonas aeruginosa* which can cause septicemia and gill necrosis under certain stress conditions^10^. Beyond this, *P. aeruginosa* plays a prominent role in the field of pathogens due to its significant impact on human health, particularly in the context of antimicrobial resistance (AMR). This pathogen is implicated in a wide range of infections, including urinary tract and pulmonary infections, which pose a major challenge for clinical management. The infections often result in the formation of biofilms, which render the bacterium insensitive to conventional antimicrobial therapies^1,2^. In addition to the bacteria mentioned above, this study focuses on the bacterial genus of *Edwardsiella (E. tarda, E. ictaluri, E. hoshinae E. piscicida* and *E. anguillarum)*, causing edwardsiellosis in both farmed and wild fish^11^. *E. anguillarum* was isolated from various aquatic species including groupers (*Epinephelus* spp.), seabream species, channel catfish (*Ictalurus punctatus*), hybrid catfish (*Ictalurus furcatus*), and tilapia (*Oreochromis* sp.)^11–13^. Investigating the pathogenicity of *E. anguillarum* isolated from a tilapia, Oh et al.^14^ found multi-focal necrotic lesions throughout the entire liver, severe congestion and signs of vasculitis causing bacteremia and septicemia in *Nile tilapia*. It is well known that several bacteria including *Edwardsiella* are affiliated with an increasing resistance to common antibiotics making them a major concern for aquaculture. Alternatives to antibiotic treatment besides the development of vaccines are therefore needed^15–19^. The bacterial pathogen’s ability to form biofilms makes antibiotic treatment and tank disinfection ineffective and complicated. However, biofilm formation can be attacked at various key positions, for example by biotechnological approaches targeting the capability of adherence, the production of extracellular polymeric substances, or by disturbing the bacterial communication. Although, the diversity of known enzymes and microbes affecting biofilms and potential pathogenic microorganisms is still rather limited, a promising source for finding novel bioactive compounds against biofilm-forming fish pathogenic bacteria is the fascinating world of microalgae and their associated bacterial communities^20,21^. Previous research showed that microalgae are surrounded by a natural community of beneficial bacteria, including interesting strategies that make this microbiota able to out-compete undesirable opportunistic bacteria such as fish pathogens or even human pathogens^21^.

Since microalgae-bacteria communities are primary producers in aquatic ecosystems, offering nutritional benefits, they provide feed sources for a sustainable aquaculture industry^22,23^. Microalgae also regulate water quality by absorbing phosphorus and nitrogen, improving water quality and thereby the survival rate of aquatic animals^24^. In addition, microalgae also have natural antibacterial activity and provide immunostimulants that have been reported to improve the innate defense mechanisms in animals, providing enhanced resistance to pathogens^25^. Microalgae developed and survived in environments where they have been exposed to a high number of microbial pathogens, including bacteria, fungi, and viruses^19^. These antagonistic interactions concerning especially the competition for inorganic nutrients have resulted in the release of algicides by the bacteria, which there upon activate the defense mechanisms of the algae^26^. These abilities have made microalgae an interesting target in research for pharmacologically active compounds against common pathogens, such as bacteria and viruses^27^. The diversity of associated bacteria within a microalgae-bacteria community is rather limited. The most frequent phyla are α-,β- & γ-*Pseudomonadota* and *Bacteroidota*^28–30^. Still, the identification of specific compounds with antibacterial activity in marine as well as in freshwater microalgae communities is challenging. Instead of looking for bioactive compounds in microalgae cultures directly, we hypothesized that metagenomic datasets of microalgae-bacteria communities encode antimicrobial compounds. Previously, we have analyzed already published datasets of microalgae and their bacterial metagenome and identified several mechanisms and biofilm-reducing candidates (Supplemental Table S1,^21^). The analysis of metagenomes of *Scenedesmus communis* (*quadricauda*), *Chlorella saccharophila* and *Micrasterias crux-melitensis,* available at IMG/MER (https://img.jgi.doe.gov), unveiled e.g., imidazolone-propionases, 6-phosphogluconolactonases, metal-dependent hydrolases, and most importantly dienelactone hydrolases (Dlh) potentially associated with quorum quenching (QQ) and significant impact on quorum sensing (QS) related to pathogen infection (^21,28,31^ Supplemental Table S1).

Therefore, in this study, we focused on the impact and characterization of a novel dienelactone hydrolase, which shows high biofilm inhibition effect on fish pathogens, e.g., *E. anguillarum.* We provide evidence that Dlh3 affects the initial attachment, essential for biofilm formation, and that *E. anguillarum* responds by the upregulation of various defense mechanisms, e.g., membrane and transport systems.

## Results

**Figure 1:**
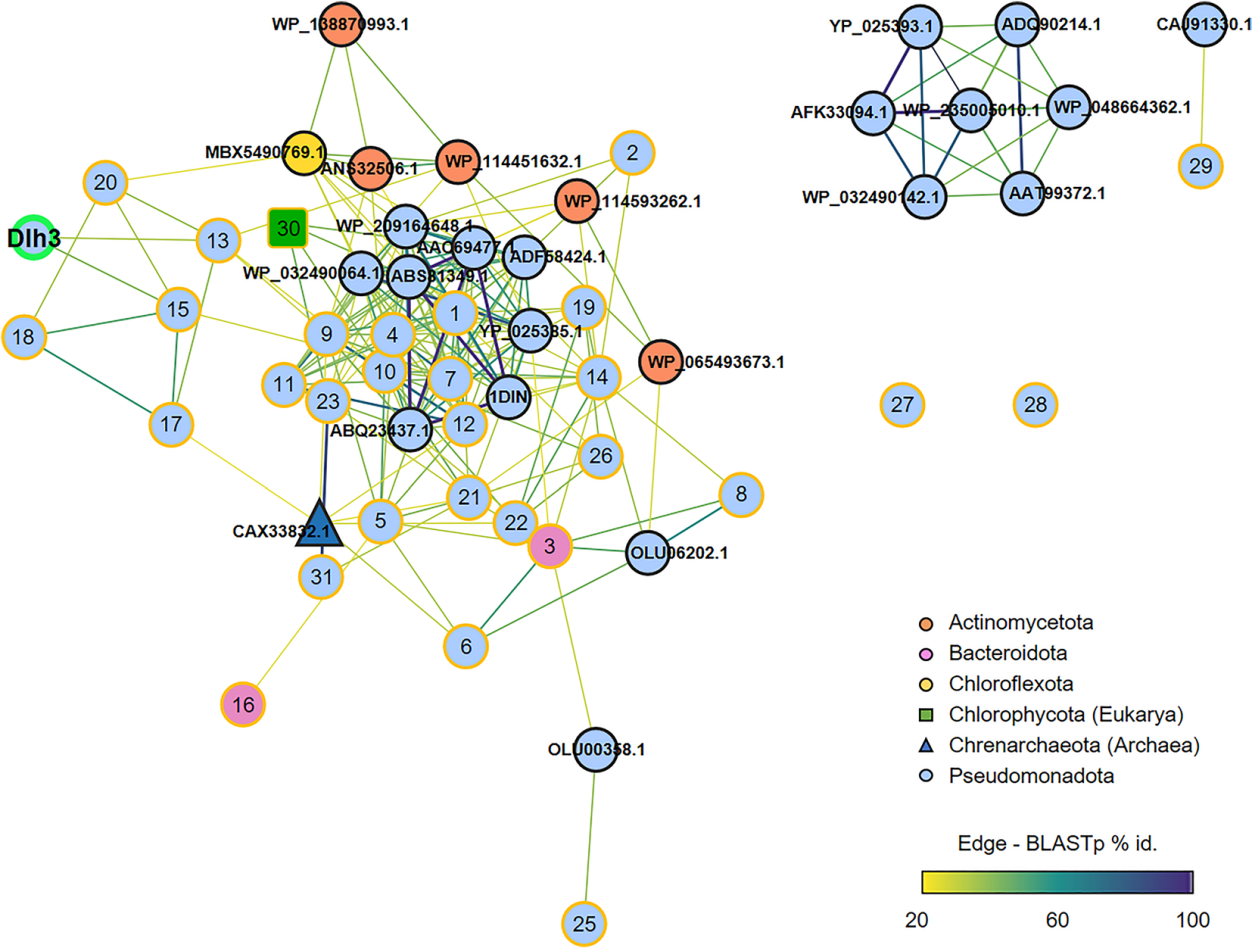
Clustering and phylogenetic analysis of predicted Dlh3 homologs in the bacterial metagenome dataset of *S. communis*^28,32^. In silico metagenomic screening for Dlhs results in 31 hits (e-value < E−10, yellow-rimmed). The amino acid sequences of Dlh3 (green-rimmed) and hits 1–31 were included in a sequence similarity network (SSN) analysis with other published Dlhs (black-rimmed). Edges connecting two nodes indicate a BLASTp e-value < 0.01, and color indicates the percentage identity. For more information on the hits, see Supplemental Table S2.

In our previously published datasets we detected a surprisingly high diversity of quorum quenching agents that are known to be involved in disturbing/interrupting the synchronization of bacteria within a community^21,28,30,32^. In total, almost 638 enzyme candidates were predicted, most of them hydrolases (number of hits: 321). The biodiversity of the most interesting dienelactone hydrolases (Dlh) was analyzed by in silico metagenomic screening of IMG datasets (Fig. 1 and Supplemental Tables S1 and S2). Clustering and phylogenetic analysis of all predicted Dlh3 homologs in the metagenome dataset of *S. communis*^28,32^ resulted in a total of 31 hits with e-values < E−10 (Fig. 1 and Supplemental Table S2), mainly associated with *Pseudomonadota*, and *Bacteroidota*. Initially, we heterologously expressed 15 potential candidates. Of these, 11 were active on *p*NP-C_8_ as substrate, and five were functionally active in a first anti-biofilm assays (this study, data not shown). Out of these Dlh3 was the most promising candidate with the best performance in the initial anti-biofilm assay and therefore selected for further investigations.

### Dlh3 possesses carboxylesterase activity and putative trans-dienelactone hydrolysing activity

**Figure 2:**
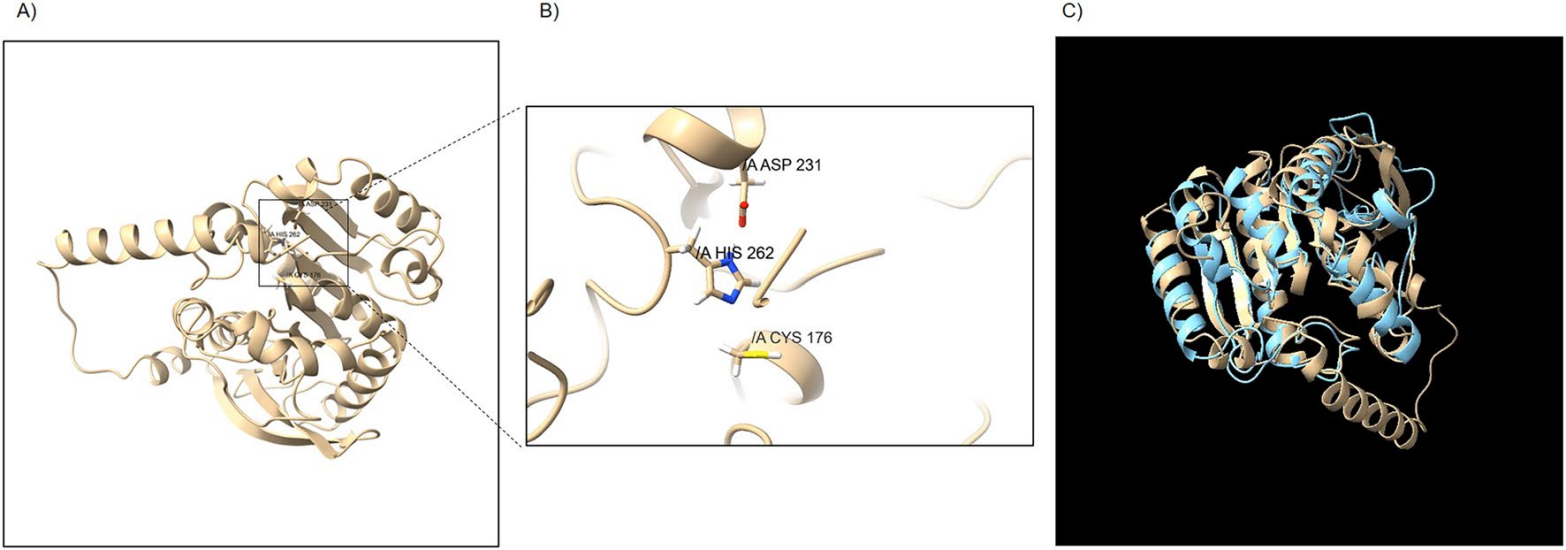
Predicted Dlh3 structure by Alphafold. (**A**) Predicted Dlh3 structure with Alphafold^35^. Dlh3 exhibits as typical α/β-protein with α-helices and β-sheets. (**B**) Movie of active site of Dlh3. Three critical amino acids (C176, D231 and H262 on Dlh3) at the active site are highlighted. (**C**) Movie of comparison of predicted Dlh3 structure (gold) with dienelactone hydrolase from *Pseudomonas* sp. B13 (sky blue, PDB ID: 1DIN,^33^). The structure match was derived with UCSF Chimera^36^. Except additional N-terminal sequence from Dlh3, the other parts including DLH-domain are fit quite well with each other. From SignalP prediction (https://services.healthtech.dtu.dk/service.php?SignalP), N-terminus of Dlh3 is TAT signal peptide (Tat/SPI, likelihood 0.948), possible cleavage site between pos. 40 and 41.

The identified Dlh3 protein comprises a Dlh domain as revealed by Pfam (E-value: 4.3e−33). Dlh3 has 73% identity to a Dlh from Alphaproteobacteria *Erythrobacter colymbi* (WP_086738354.1, E-value: 5e−104). Its predicted 3-D structure shows that Dlh3 exhibits as typical α/β-hydrolase with 8 α-helices and 8 β-sheets (Fig. 2A). When compared to the Dlh crystal structure from *Pseudomonas knackmussii* (previously named as *Pseudomonas* sp. B13; PDB ID: 1DIN)^33^, the Dlh domain of Dlh3 fits quite well with this reference structure (Fig. 2C). According to the sequence- and structure-alignments, Dlh3 contains typical catalytic triad Cys-His-Asp (C176, D231 and H262 on Dlh3) in the active site (Fig. 2B). Additionally, Dlh3 comprises one additional α-helix at its N-terminus, which is likely to be a TAT signal peptide (Tat/SPI, likelihood 0.948) as indicated by SignalP. This signal peptide in Dlh3 may be involved in the translocation of Dlh3, resulting in the regulation of quorum sensing (QS) signals like some reported lactonases^34^.

The *dlh3* gene was amplified by PCR from the metagenomic DNA of a *S. communis* culture and cloned into the vector pET21a+. After overexpression in *E. coli* Rosetta-gami™ 2(DE3), a 32-kDa His-tagged Dlh3 protein could be purified. Its hydrolase activity was analyzed with *p*NP-C_8_ as substrate under different conditions (Supplemental Fig. S1). At 37 °C and 55 °C Dlh3 exhibited its highest activity in a buffer with pH 8.0 (Supplemental Fig. S1A,B). The activity was strongly reduced at 65 °C. The enzyme also hydrolyzed *p*NP-C_8_ at moderate temperatures (i.e., at 22–42 °C as shown in Supplemental Fig. S1C). These results indicated that Dlh3 is a thermostable enzyme and exhibits carboxylesterase activity.

The Dlh family can be divided into three types according to their substrate specificities (Supplemental Fig. S2^37^): Type I Dlhs possess *trans*-dienelactone hydrolysing activity (e.g., Dlh from *Saccharolobus solfataricus*^38^), Type II Dlhs only *cis*-dienelactone hydrolysing activity (e.g., Dlh from *Pseudomonas cepacia*^39^), while Type III Dlhs are active against both *cis*- and *trans*-dienelactones (e.g., 2 Dlhs from *Cupriavidus necator* JMP134^40^). The phylogenetic tree implies that Type III Dlhs are clustered into two sub-groups, i.e., group A Dlhs exhibit higher catalytic rate to *trans*-dienelactone, such as Dlh from *Pseudomonas knackmussii* (PDB ID: 1DIN)^33^ and TfDEI from *C. necator* JMP134^40^, while group B Dlhs show higher rate of *cis*-dienelactone conversion, e.g., TfDEII from *C. necator* JMP134^40^. The phylogenetic tree also shows that Type III Dlhs from group B have closer evolutional relationship to Type II Dlhs and group A Dlhs are closer to Type I Dlhs. Our Dlh3 identified from *S. communis* associated microbial communities is phylogenetically clustered within Type I Dlhs, indicating that it may only be active on *trans*-dienelactone (Supplemental Fig. S2).

### Effect of Dlh3 on biofilm formation of various fish pathogens

**Figure 3:**
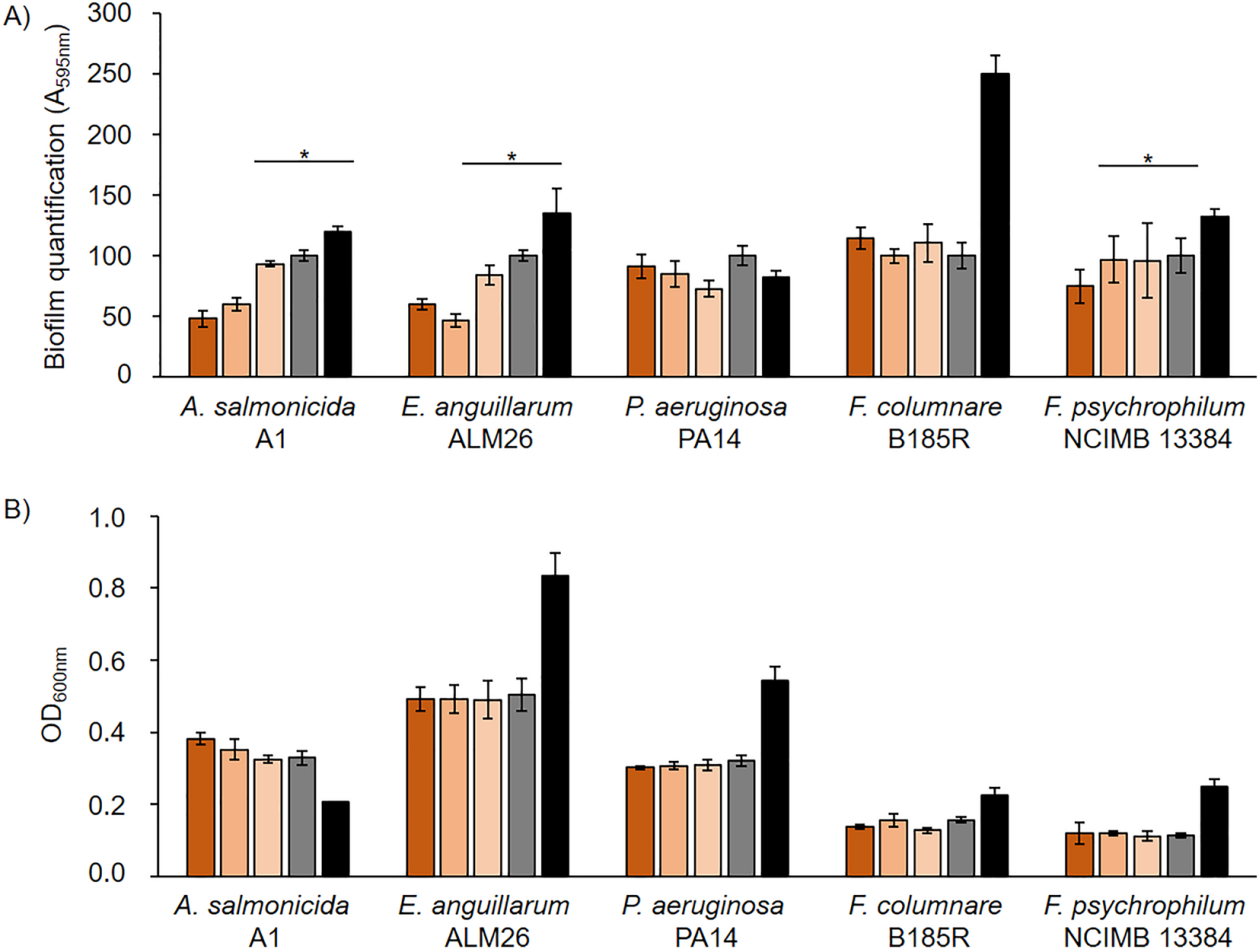
Static biofilm assays. (**A**) antibiofilm tests, 48 h of incubation at 28 °C, stained with crystal violet 0.5% Biofilm producer: *Aeromonas salmonicida* A1, *Edwardsiella anguillarum* ALM26, *Pseudomonas aeruginosa* PA14, *Flavobacterium columnare* B185R*, F. psychrophilum* NCIMB 13384. Protein: dienelactone hydrolase (Dlh3). The dienelactone hydrolase was overexpressed in *E. coli* Rosetta-gami™ 2 (DE3), purified, dissolved in 1×PBS and added in the following concentrations: color key: brownish for 1×  = 0.5 mg/mL, orange: 0.1×  = 0.05 mg/mL, and beige colored: 0.001×  = 0.005 mg/mL, grey: PBS control, black: bacteria media control. Statistical analyses were subjected to a paired sample *t*-test and the *p* value was referred to define if the samples are significantly different in the presence of the enzyme Dlh3 compared to the PBS control. Significant biofilm reduction is marked by stars (significance level *p* value ≤ 0.05). (**B**) Measurement of optical density (OD_600nm_) after 48 h of incubation at 28 °C.

For its mode of action in hydrolyzing lactone rings, Dlh3 is assumed to act as a quorum quenching agent. Here, the effects of Dlh3 on various fish pathogens (*Aeromonas salmonicida*, *Edwardsiella anguillarum*, *Pseudomonas aeruginosa*, *Flavobacterium columnare* and *Flavobacterium psychrophilum*, and two different strains of *Yersinia ruckeri*) was evaluated by performing static biofilm assays with crystal violet (CV) staining for detecting all attached bacteria and extracellular matrix (Fig. 3) and BacTiter-Glo™ based assessment of metabolic activity (Supplemental Fig. S3), in addition to a flow-based biofilm assay on the selected strain of *E. anguillarum*.

A statistically significant reduction in biofilm formation, compared to the PBS control, was detected for *A. salmonicida* (44.2% ± 6.2%), *E. anguillarum* (41.5% ± 3.1%) and *F. psychrophilum* (21.4% ± 4.0%) after treatment with 0.5 mg/mL Dlh3 (based on a *p* value ≤ 0.05). Addition of 0.05 mg/mL Dlh3 achieved a reduction in biofilm formation for *A. salmonicida* (35.9% ± 3.3%), *E. anguillarum* (54.5% ± 6.5%) and *P. aeruginosa* (19.1% ± 2.4%), all statistically significant. This result was consistent for both biofilm quantification methods. Treatment with a one hundred times diluted protein solution (0.005 mg/mL Dlh3) only showed an effect in reducing *P. aeruginosa* biofilm. Bacterial growth in the supernatant was mostly not significantly affected by Dlh3. Only growth of *E. anguillarum* and *Y. ruckeri* CSF007 was reduced in the presence of the enzyme, by 23% and 35%, respectively. The most constant results could be achieved with the highest enzyme concentration tested, which was the selected concentration in the following assays.

### Pronounced inhibition of biofilm formation by *Edwardsiella anguillarum*

Based on the promising results of up to 54.5% *E. anguillarum* biofilm inhibition by Dlh3, this bacterium was the focus in the following analyses to uncover the underlying mechanisms and possible pathways.

#### Reduced cell numbers in confocal imaging analysis

**Figure 4:**
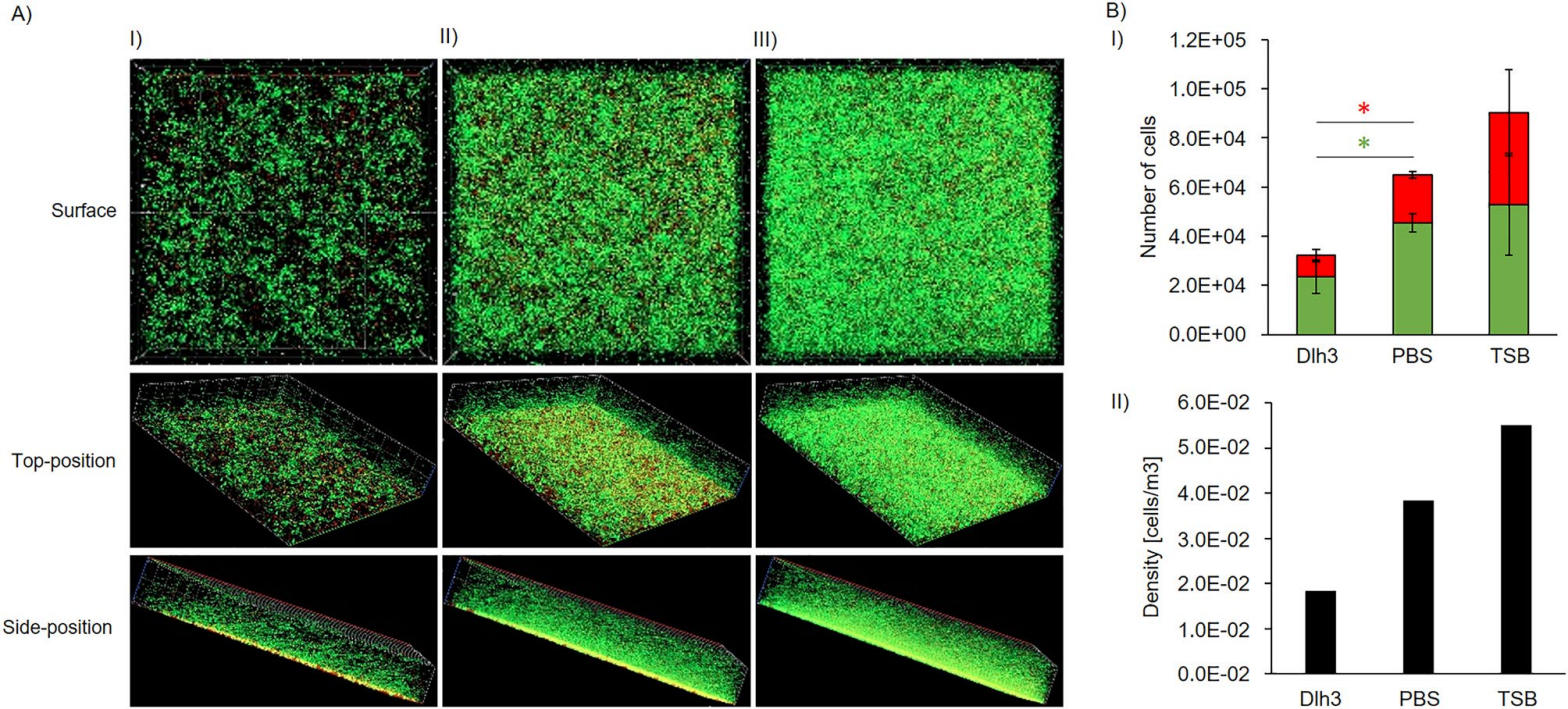
Confocal microscope images of static biofilms. (**A**) Confocal microscopy, biofilm producer: *Edwardsiella anguillarum* ALM26. Incubation with test liquids at 28 °C for 48 h. Staining: SYTO 9 (green fluorescence) for living cells, Propidium iodide (red fluorescence) for dead cells, x-axis 200 µm, y-axis 200 µm, z-axis 20 µm, z-stacks of 80 slides. I) dienelactone hydrolase 3 (Dlh3), II) Control: PBS, III) Control: TSB. Shown is surface of z-stack, top-position and side-position. (**B**) Calculation of cell numbers (I) and density (II) of the biofilm. Performed with MatLab/BiofilmQ (https://drescherlab.org/data/biofilmQ/docs/). For the determination of the significance of certain data, a statistical test was used. The number of living and dead cells, the biofilm thickness, and the biofilm density were subjected to a paired sample *t*-test and the *p*-value was assigned to determine if the two samples were significantly different from each other. Significant reduction of cell numbers is marked by stars, red star = dead cells, green star = living cells.

Confocal imaging analysis was performed to evaluate biofilm characteristics beyond the quantitative biofilm assays, and to obtain absolute cell numbers in observed biofilms as well as portions of living and dead cells. The results from the CV and BacTiter-Glo™ -based biofilm assays were confirmed by confocal imaging of *E. anguillarum* biofilm treated with Dlh3 (Fig. 4 and Supplemental Fig. S4). A reduction of 51% in total cell number was observed compared to the PBS control. The number of living cells was reduced by 48% (*p* value 0.05), while the number of dead cells was 56% (*p* value 0.04) lower when treated with 0.5 mg/mL Dlh3. The biofilm structure changed significantly by appearing more porous and pitted. In contrast to the homogenous character of the untreated biofilm, the Dlh3-treated biofilm showed patches of higher and lower density up to areas free from attached bacteria. The height of the Dlh3-treated biofilm was considerably lower than in the PBS control. A calculated reduction of the biofilm density by 52% compared to the control confirmed the visual impression and confirmed the disturbing effect of Dlh3 on the organization of the unicellular bacteria in the biofilm.

#### Biofilm inhibition in a flow-based test

Fluorescence microscopy images confirmed that Dlh3 inhibited biofilm formation by *E. anguillarum* under flow conditions at both 0.5 and 0.25 mg/mL of the enzyme compared to the untreated control (Supplemental Fig. S4). The highest inhibition was seen at treatment with 0.5 mg/mL Dlh3. No dead biofilm cells were detected, the flow of medium probably detached dead cells from the biofilm. These data were congruent with the confocal imaging analyses described above, the influence of Dlh3 leads to a structurally changed cell layer in *E. anguillarum*, which may influence the force of adherence to the surface.

### Transcriptome analysis identifies highly active genes of *Edwardsiella anguillarum* related to transport systems

**Figure 5:**
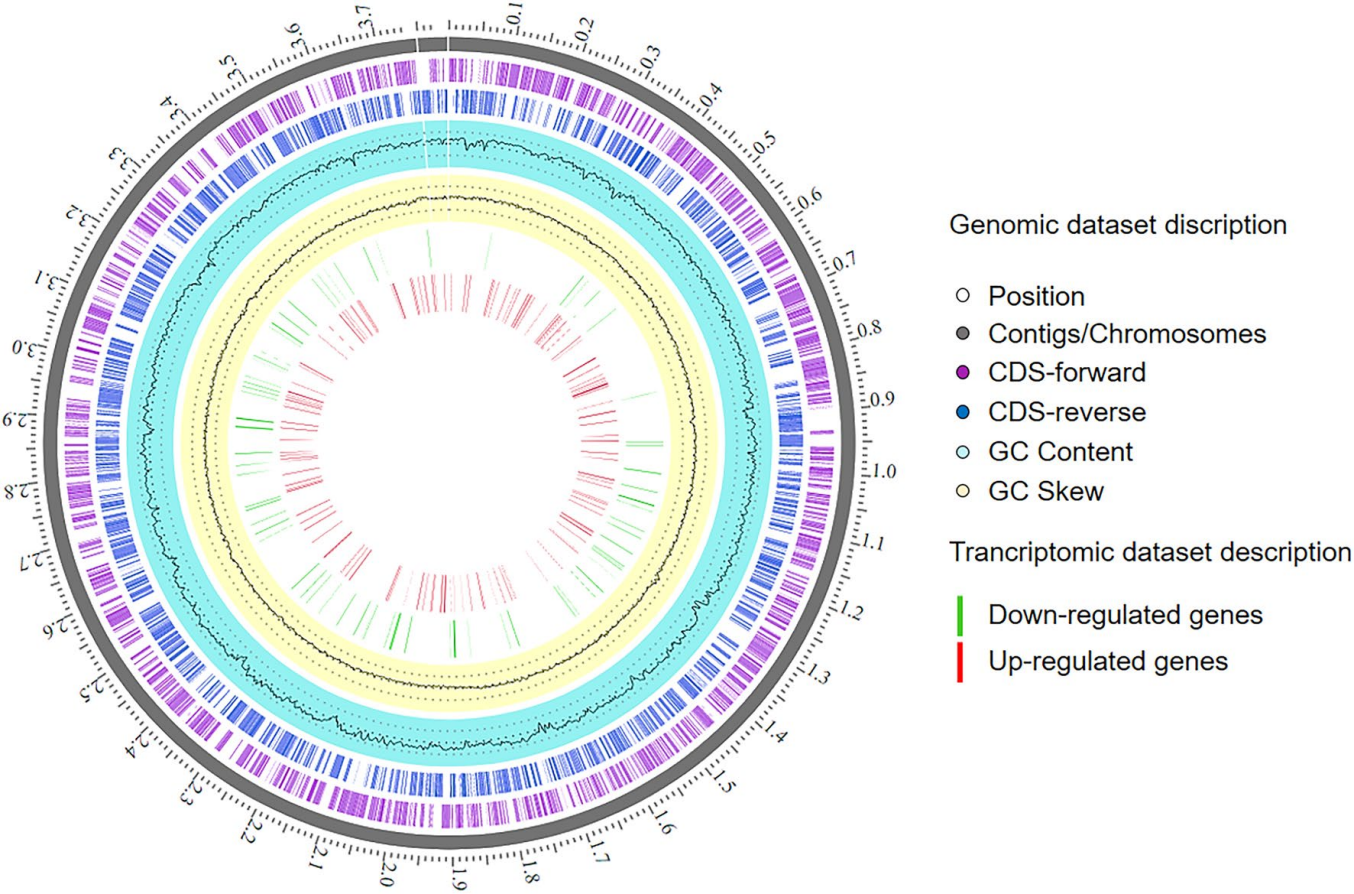
Circular mapping. Transcriptome analysis and circular genome map of transcriptome datasets of *Edwardsiella anguillarum* ALM26 after treatment with 0.5 mg/mL Dlh3. The circular mapping was generated using PATRIC^41^. Moving inward, the subsequent two rings show CDSs in forward (magenta) and reverse (blue) strands. Cyan and yellow plots indicate GC content and a GC skew [(GC)/(G + C)]. Transcriptomic dataset description in the presence on Dlh3 red: up-regulated genes, green: down-regulated genes.

**Figure 6:**
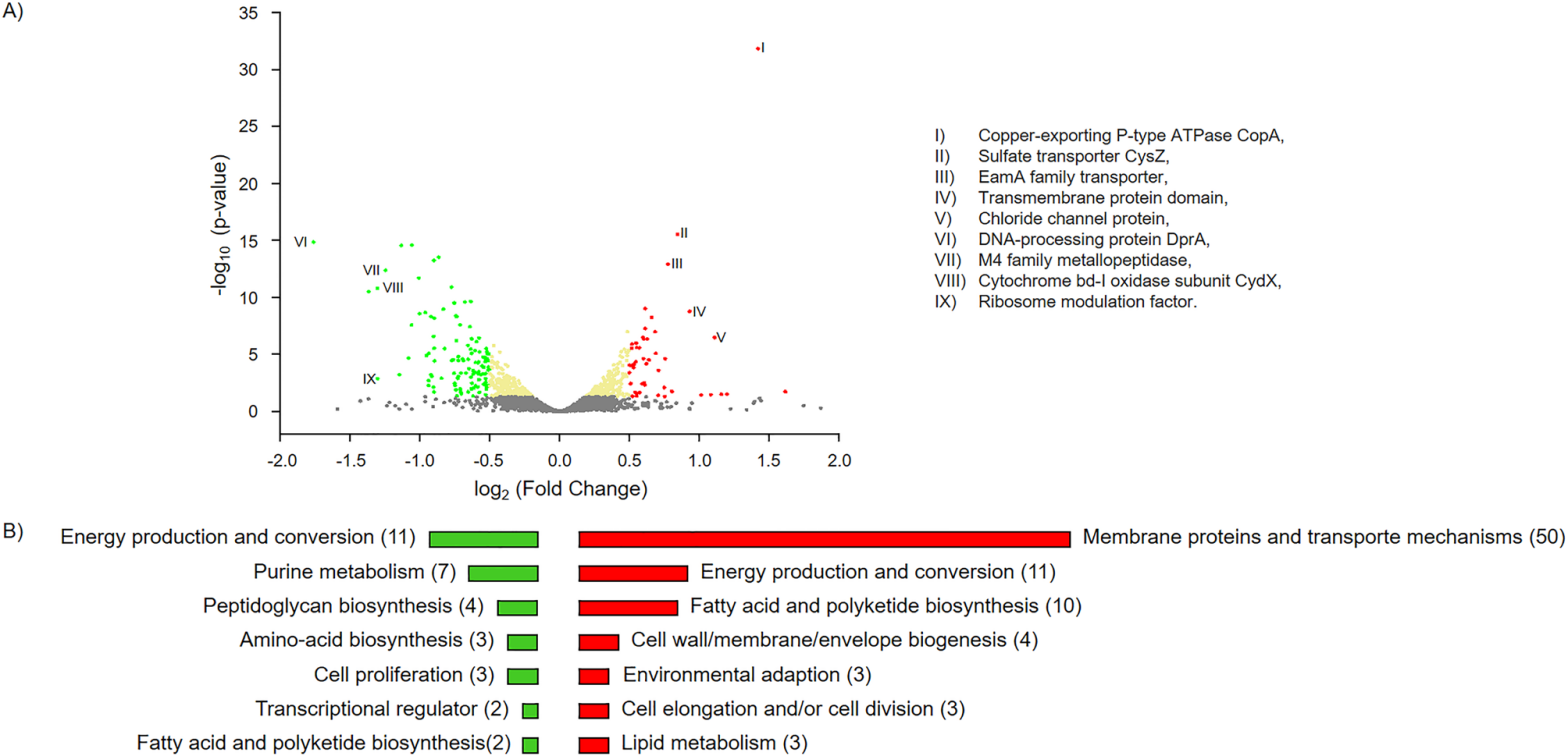
Differentially expressed genes (DEGs). DEGs in *Edwardsiella anguillarum* ALM26 co-cultured in the presence on 0.5 mg/mL Dlh3 compared with control dataset. (**A**) Volcano plot of transcriptome data includes all measurable genes, and highlighting up and down regulated genes in *E. anguillarum* encoding the following proteins: (I) copper-exporting P-type ATPase CopA, (II) sulfate transporter CysZ, (III) EamA family transporter, (IV) transmembrane protein domain, V) chloride channel protein, VI) DNA-processing protein DprA, (VII) M4 family metallopeptidase, VIII) cytochrome bd-I oxidase subunit CydX, (XI) ribosome modulation factor. (**B**) Function profile of differentially expressed genes in *E. anguillarum* is presenting the groups of most active genes. Total number of genes are shown in brackets. Color key: red = up-regulated genes, green = down-regulated genes.

To unravel the potential inhibitory mechanism of Dlh3, we investigated the transcriptome of *E. anguillarum* in the presence and absence of the enzyme. In summary, these data elucidate the gene expression in response to Dlh3, with potential implications for biofilm formation. The sequencing of the mRNA resulted in a minimum of 9.53 million sequences with an average length of 75 bp and a GC content of 55%. Overall, a total of 3297 expressed genes were identified from the dataset (Supplemental Table S3). The circular genome mapping of the datasets was generated to the reference genome of *E. tarda* EIB202 (NC_013508.1) and visualized in Fig. 5. The up- and down-regulated genes were presented in the inner rings in red and green, respectively (Fig. 5) and summarized in Supplemental Table S3. To quantify the differential gene expression of *E. anguillarum* under the influence of Dlh3 log_2_ (Fold change) values were calculated for all identified genes indicating up or down regulation of respective functions. Their significances were plotted in a Volcano plot and in a functional profile analysis (Fig. 6).

In addition to the generally regulated metabolic genes, e.g., for amino acid, carbohydrate, lipid and nucleotide transport and metabolism, transcription and translation, as well as signaling transduction mechanisms; we could identify genes linked to cell motility, secretion systems, biofilm formation, quorum quenching and quorum sensing mechanisms. The analysis shows high expression levels for flagellar biosynthesis, type III secretion effectors, and channel proteins. The analysis showed that the most significant up-regulated genes are affiliated with the transport mechanisms and membrane proteins, including cell division proteins, antiporter membrane proteins (e.g.; ABC, CysZ, EamA family, outer membrane porin, OmpA family; Protein translocase membrane subunit SecG; inner membrane protein RarD; UPF0126 inner membrane protein YicG). We discovered the highest upregulation of gene expression for the copper-exporting P-type ATPase *copA*. Copper as cofactor in proteins involved in electron transport and iron trafficking has a high importance in cellular metabolism of bacteria^42,43^. Proper regulation of intracellular copper levels is vital, as high levels of copper can induce cell damage or death^44^. As reaction to host- or environmental mediated copper intoxication, bacteria can express a wide range of efflux systems to maintain metal ion homeostasis, e.g. the P-type ATPase family. CopA is regulated by the MerR-family regulator CueR which is activated by Quorum Sensing signaling via acetyl-HSL autoinducer molecule^45^. This P-type ATPase CopA is known to modulate oxidative stress resistance, potentially contributing to increased tolerance to toxic metal species in biofilms induced by Quorum Sensing pathways^44–47^. Our findings suggest that comparable mechanisms exist for further molecules or elements since the majority of the significantly upregulated identified genes are involved in transport mechanisms or are membrane proteins (50 genes) e.g. chloride channel protein and sulfate transporter *cysZ.* Beside this, in the presence of Dlh3, upregulation of *N*-acyl-L-homoserine lactone synthase gene activity was identified, the product of which is *N*-acyl-L-homoserine lactone, a signaling molecule involved in quorum sensing.

Down-regulated genes were mainly found to encode for energy production and conversion mechanisms (11 genes), fatty acid and polyketide biosynthesis (2 genes), amino-acid biosynthesis (3 genes), cell wall biogenesis (3 genes), and peptidoglycan biosynthesis (4 genes). Most relevant are the DNA-processing protein DprA, M4 family metallopeptidases, Cytochrome bd-I oxidase subunit CydX, and Ribosome modulation factors.

### Cell toxicity assay show no influence on cell line CHSE-214

In order to consider a possible biotechnological application of candidates in the aquaculture sector, the compatibility with living cells has to be taken into account. For this, in vitro cytotoxity of Dlh3 was studied in CHSE-214 cells (Chinook Salmon Embryo). At all tested concentrations (2.55–244 µg/mL), Dhl3 did not markedly affect cell viability relative to cells exposed to PBS (Supplemental Fig. S5). We could prove, that in a fish cell model the viability was not impaired by Dlh3.

## Discussion

Sustainability is a major goal in aquaculture farming with animal health and welfare being a high priority and need. In addition to vaccination, new strategies to treat bacterial infectious diseases and biofilms other than antibiotics or chemotherapeutics are of great interest. In this study, we focused on several fish pathogens including *A. salmonicida*, *E. anguillarum*, *P. aeruginosa*, *F. columnare* and *F. psychrophilum*, all important causatives of severe infections like furunculosis, edwardsiellosis, hemorrhagic septicemia, columnaris disease and cold-water disease, respectively^10,14,48–50^. One of the first steps of the biofilm forming pathway of an individual bacterium is the secretion of autoinducer molecules [most often types of acetyl homoserine lactones (AHL)] into the bacteria’s environment. Once the production of these molecules by numerous individuals passes a certain threshold, they bind to bacterial transmembrane receptors and activate a DNA-binding element to differentiate gene expression in order to attach on surfaces and induce virulence. The AHL-LuxR quorum sensing system was intensively studied in *P. aeruginosa*^51^. Homologues of autoinducer synthases, receptors and DNA-binding elements were identified in a wide range of bacterial species. Within this study we focused on disruption of this first communication signal between planktonic, unicellular bacteria by adding a novel dienelactone hydrolase to growing bacteria.

The successful reduction of biofilm formation of *A. salmonicida* and *E. anguillarum* by 40–50% should be reasoned in the use of LuxRI homolog systems for quorum sensing in both bacteria. Swift et al.^52^ found genes coding for a quorum sensing signal generator and a response regulator named AsaRI for *Aeromonas salmonicida* as well as the AHL homolog *N*-butanoyl-_L_-homoserine lactone (BHL). Comparable findings were made for *E. tarda*, a closely related species to *E. anguillarum*. Morohoshi et al.^53^ sequenced the quorum sensing genes, *luxI* homolog (*edwI*) and luxR homolog (*edwR*) as well as two kinds of AHLs produced by *E. tarda* which seemed to be N-hexanoyl-L-homoserine lactone (C6-HSL) and N-heptanoyl-L-homoserine lactone (C7-HSL). Biofilm formation and virulence factors therefore seem to be under the regulation of a lactone ring-based AHL autoinducer, which makes them susceptible for the cleavage mechanism of dienelactone hydrolase. It may only be speculated why *P. aeruginosa* showed minor reduction in biofilm formation, compared to *A. salmonicida* and *E. anguillarum*, as it belongs to the most extensively studied cell-to-cell communication systems with known (AHL)-dependent regulatory circuits^51^. At least, *P. aeruginosa* uses three different autoinducer molecules. Besides AHL it synthesizes cyclic dipeptides and quinolone, which may replace each other and lead to lower susceptibility towards a specific anti-AHL agent^54^. The quorum sensing systems of *Flavobacteria* as members of the phylum *Bacteroidota*, are still under investigation. LuxRI homologs in respective taxa are not described in literature yet, which may make *Flavobacteria* less susceptible to dienelactone hydrolase activity.

Our transcriptomic data revealed altered regulation of transport systems in the presence of Dlh3 but interestingly not the virulence associated type III, IV, V secretion systems. Neither were further biofilm forming or virulence genes significantly upregulated like flagellar or pili associated genes, polysaccharide associated genes or glycosyltransferases. We observed upregulations in functions connected to self-defense like upregulation of membrane proteins and genes for export mechanisms and transporters. Since dienelactonases act as hydrolases of the quorum sensing molecule AHL, the transcriptome data give an indication, that *Edwardsiella* probably does not get the usual amount of autoinducer, to feel safe in a community, to settle down and start attacking a host. It seems like the sensing of other bacteria in its environment is impaired and that *Edwardsiella* is still in a mode of unicellular, planktonic organism. This hypothesis is supported by the finding of upregulation of quorum sensing associated genes, especially of the L-homoserine lactone synthase.

In summary, the results showed the remarkable potential of microalgae-bacteria communities’ genetic resources and their biological antimicrobial activity properties exemplified by reduction in biofilm formation by *E. anguillarum*. To be more specific, the current study gives a detailed insight into the effects of the metagenome derived dienelactone hydrolase Dlh3, to have measurable reductive effects on fish pathogens´ biofilm formation. The microscopic and transcriptome dataset clearly demonstrated the effect of Dlh3 on the fish pathogen *E. anguillarum* by reducing the formation of biofilms, possibly through interference with quorum sensing system of the bacterium.

The substrate specificity of Dlhs and antibiofilm activity against particular bacterial strains highlights the potential for targeted interventions aimed at disrupting specific metabolic pathways or physiological processes critical for biofilm formation and maintenance. Understanding these characteristics can pave the way for the development of more precise and effective strategies for combating biofilm-related issues and controlling the growth and persistence of problematic bacterial populations e.g., in aquaculture settings. In addition, the absence of toxic effects on growth of fish cell line CHSE-214 opens up for a putative biotechnological application of Dlh3.

Nevertheless, the potential for application of the results from the present study in aquaculture settings needs further studies, with respect to both fish welfare and possible ways for product utilization. Especially, the antibiofilm activity within multispecies biofilms, commonly found in aquaculture systems, presents a complex interplay of interactions among different microbial species. While biofilms themselves are often associated with pathogenicity and resistance to antimicrobial agents, the presence of multiple species within a biofilm community can lead to diverse and dynamic interactions that influence biofilm formation, maintenance, and susceptibility to antibiofilm strategies. Future work should address further bioactive molecules to target a broader spectrum of fish pathogens, face the usage of different autoinducer molecules and answer local demands. The AHL class of autoinducers is susceptible not only for cleaving the lactone ring but also for cleaving the acyl side chain by acylases or oxidation/reduction of the acyl side chain by oxidoreductases. A number of novel enzymes could be applied in a concerted way (e.g., use for pre-treatment and cleaning of fish or surface preparation of tanks), each of them low dosed and neutral to farmed animals and environment, but in the way of targeting various aspects of biofilm formation and avoiding quick adaptation and resistance in the pathogenic bacteria.

## Material and methods

### Bacterial strains, microalgae, and culture conditions

Bacterial and microalgal strains, as well as plasmids are listed in Supplemental Table S4. Protein overexpression was performed in *E. coli* Rosetta-gami™ 2(DE3) (Merck, Darmstadt, Germany), which was cultured in lysogeny broth (LB,^55^) at 37 °C. The fish pathogens *E. anguillarum* (DSMZ No: ALM26) and *A. salmonicia* (DSMZ No. A1) were cultivated in trypticase soy broth (TSB) and nutrient broth (NB,^55^), respectively, at 28 °C. The fish pathogens *Flavobacterium columnare* (strain B185R) and *F. psychrophilum* (strain NCIMB 13384) were cultivated in tryptone yeast extract salts broth (TYES^56^) medium at 15 °C or 22 °C. *P. aeruginosa* (strain PA14) was cultivated in LB medium at 37 °C. *Yersinia ruckeri* (NCIMB 1315 and CSF007) was grown in LB medium at 28 °C. When required, antibiotics were used at the following concentrations: ampicillin 100 µg/mL; chloramphenicol 25 µg/mL.

Microalgae *S. communis* (previously named as *Scenedesmus quadricauda* MZCH10104) was obtained from the collection of algae at the University of Hamburg, Germany (http://www.biologie.uni-hamburg.de/bzf/zeph/zephsvcke.htm). The cultivation of the alga was carried out in Kessler and Czygan-media with minor modifications^28,57,58^ at 22 °C with a final concentration of 4% CO_2_ at a natural light intensity as described in Krohn-Molt et al.^28^.

### Sequence and structural alignments

A profile Hidden Markov Model (HMM) search was performed as described previously^59^. Shortly, the curated Dlh model (PF01738.21) from the InterPro/Pfam database^58,60^ was applied with hmmsearch from the HMMER v.3.3 software package^61^ on all CDS from the metagenome. The cut-off was set at an e-value < E−10. The phylogenetic affiliation of each potential Dlh was inferred from the best DIAMOND BLASTp hit in the non-redundant protein database of NCBI^62^. Subsequently, a DIAMOND database was constructed together with all reference sequences in Supplemental Fig. S2 and “all against all” alignments were performed with DIAMOND BLASTp v.2.0.15^63^, reporting only alignments with e-value < 0.01. Visualization of the Sequence Similarity Network (SSN) was conducted in Cytoscape v.3.9.1^59^ applying the “Prefuse Force Directed Layout (none)” as described elsewhere^64^. The edge color was mapped against the sequence identity.

The sequence of the best performing dienelactone hydrolases (Ga0078192_1052715_Dlh3) was analyzed with Pfam protein families database^60^ and SignalP 6.0^65^. The sequences of Dlhs were compared with the non-redundant protein database of NCBI by using BLASTP^66^. The phylogenetic tree was constructed with MEGA X^67^ based on the maximum-likelihood method and JTT matrix-based model^68^ with 1000 bootstraps after multiple sequence alignments (MSA) with T-Coffee^69^.

The structure of Dlh3 was modelled with AlphaFold v2.1.0 on Colab^35^, and the structure was visualized and analyzed with UCSF Chimera X^36^.

### Molecular cloning, expression and purification of recombinant Dlh3

The 0.90 kb BamHI–XhoI DNA fragment, containing metagenome-derived Dlh3 sequence, was amplified from the metagenome of *S. communis* culture by PCR (using primers 5′-CGGGATCCATGTGCGATGAAAGCAAGCTTGAC-3′ and 5′-CCGCTCGAGCGCCAGCGCCCCCTT-3′) and cloned into the *Bam*HI and *Xho*I sites of vector pET21a+(Merck, Darmstadt, Germany). The recombinant plasmid designated pET21a+::*dlh*3 was used to transform *E. coli* Rosetta-gami™ 2(DE3) (Merck, Darmstadt, Germany). The obtained construct was verified by sequencing (Microsynth, Göttingen, Germany).

Heterologous expression of the His-tagged Dlh3 was induced at 22 °C overnight with 0.5 mM isopropyl-b-D-thiogalactopyranoside (IPTG). The purification of the enzyme was performed at 4 °C using Protino^®^ Ni-NTA Agarose (MACHEREY-NAGEL, Düren, Germany) according to the manufacturer’s instructions in a lysis buffer [50 mM NaH_2_PO_4_ pH 8.0, 300 mM NaCl, 10 mM imidazole, 0.1 mM phenylmethylsulfonyl fluoride (PMSF), 1 mM dithiothreitol (DTT)]. Then the agarose was washed with wash buffer (50 mM NaH_2_PO_4_ pH 8.0, 300 mM NaCl, 20 mM imidazole, 0.1 mM PMSF, 1 mM DTT). The protein was finally eluted with elution buffer (50 mM NaH_2_PO_4_ pH 8.0, 300 mM NaCl, 250 mM imidazole, 0.1 mM PMSF, 1 mM DTT). The eluted protein was dialyzed in Roti^®^ cell PBS buffer (Carl-Roth, Karlsruhe, Germany) at 4 °C and sterile filtered (pore size 0.20 µM) before application in various assays. The protein concentration was determined using a nanophotometer (Implen, Munich, Germany) with a molar extinction coefficient of 43,555 M^−1^ cm^−1^.

### Enzyme activity assays

The carboxylesterase activity of Dlh3 was analyzed with 4-nitrophenyl octanoate (*p*NP-C_8_) as substrate. The colourless substrate releases chromogenic *para*-nitrophenol (yellow) when the ester bond is hydrolyzed, which can then be detected photometrically at a wavelength of 405 nm^38^. Each reaction was performed with 2 mM *p*NP-C_8_ as substrate in 100 mM potassium phosphate buffer with different pH. The reactions were incubated at different temperatures for 1 h and stopped by adding Na_2_CO_3_ with final concentration of 200 mM. Afterwards, the samples were centrifuged at a speed of 16,000× *g* for 3 min. 150 µL supernatant was used to record the absorbance at 405 nm in a plate reader (Biotek, Winooski, United States). All samples were measured in triplicates. To determine the optimal temperature, samples were incubated at 22 °C, 28 °C, 37 °C, 42 °C, 55 °C, and 65 °C in 100 mM potassium phosphate buffer with pH 8.0. The influence of pH conditions on the activity was measured in 100 mM potassium phosphate buffer with pH 6.0, 6.5, 7.0, 7.5, and 8.0 at either 37 °C or 55 °C.

### Static biofilm inhibition tests

Biofilm formation was quantified using two alternative methods, the crystal violet (CV) method to detect both living and dead bacteria based on staining extracellular matrix polymers, and the ATP-based BacTiterGlo^®^ method to quantify metabolic activity. The CV-based protocol was performed as follows: The bacteria from an overnight preculture were adjusted to OD_600nm_ = 0.05 with TSB medium (Trypticase Soy Broth, Merck). 50 µL of the diluted bacterial culture were pipetted in six replicates for each condition into a 96-well plate (Thermo Fisher Scientific, flat bottom, Nunclon Delta surface, Waltham, MA). 50 µL of test liquid were added (protein solutions: 0.5 mg/mL = 0.01563 µmol/mL, 0.05 mg/mL, 0.005 mg/mL), 1 × PBS, TSB). Plates were incubated at 28 °C for 48 h. After incubation, the OD_600nm_ of the supernatants was measured in a Microplate Reader (Synergy HT, BioTek, Agilent Technologies, Santa Clara, United States). For staining all wells were washed three times with 200 µL of H_2_O_bidest_, then 150 µL of 0.5% Gram ‘s crystal violet solution (hexamethyl pararosaniline chloride, ethanol, phenol, Merck KGaA, Darmstadt, Germany) were pipetted in each well and the plate was incubated for 10 min at room temperature. After incubation, plates were washed three times in a water bath of tap water, dried at 37 °C for 30 min and 150 µL of 33% acetic acid were added to each well. The plates were incubated another 10 min at room temperature and then biofilms were quantified at wavelength λ = 595 nm with a Microplate Reader. The ATP-based protocol was performed as follows: On day one, precultures of all strains were prepared in their respective liquid growth medium. The next day, precultures were diluted to the following optical densities: *E. anguillarum* ALM26 (OD_600nm_ = 0.1 in 40% TSB), *A. salmonicida* strain A1 (OD_600nm_ = 0.1 in 40% NB), *Y. ruckeri* NCIMB1315 and CSF007 (OD_600nm_ = 0.2 in 40% LB), *F. psychrophilum* NCIMB13384 (OD_600nm_ = 0.4 in TYES), 75 µL of these diluted precultures were transferred to white 96-well white/clear bottom plates with TC surface (Thermo Fisher Scientific, Waltham, MA, USA) in four parallels per test substance or control. These were mixed with the same volume of either growth medium, PBS or Dlh3 protein solution in PBS. Thereby, growth media are reduced in nutrient strength, which is known to promote biofilm formation. Plates were covered with a lid, sealed with parafilm, and incubated in a humid atmosphere under static conditions at either 28 °C for one day (*E. anguillarum*), 28 °C for two days (*Y. ruckeri* and *A. salmonicida*) or 15 °C for three days (*F. psychrophilum*). To analyze the effect of test substance on bacterial biofilms, 100 µL of supernatant were transferred to Nunc^®^ 96-well polystyrene plates with flat bottom (Sigma-Aldrich) to determine OD_600nm_. Biofilms attached to the bottom of the plates were washed four times with PBS and a final transfer of 100 µL PBS to each well. At this point, biofilm formation was visually inspected using the EVOS FL Auto Imaging system (LifeTechnologies™). Further, the biofilms were relatively quantified using the BacTiter-Glo™ Microbial Cell Viability Assay (Promega) according to the manufacturer's instructions.

### Confocal imaging analysis

The bacteria from an overnight preculture were adjusted to OD_600nm_ = 0.05 with TSB medium.100 µL of the diluted bacteria culture were pipetted into each well of an eight-well *µ*-slide (Ibidi USA Inc., Fitchburg, WI). 100 µL of test liquid were added to the bacteria culture (protein solution, PBS and TSB medium). Incubation took place at 28 °C for 48 h. After incubation all supernatants were discarded and 150 µL of staining solution were added, using the LIVE/DEAD BacLight bacterial viability kit (Thermo Fisher Scientific, Waltham, MA). Visualization of the biofilms was performed with a confocal laser scanning microscope (CLSM) Axio Observer.Z1/7 LSM 800 (Carl Zeiss Microscopy GmbH, Jena, Germany) and a C-Apochromat 63×/1.20W Korr UV VisIR objective. The analysis of the CLSM images and three-dimensional reconstructions were done with ZEN software (version 2.3; Carl Zeiss Microscopy GmbH). Living and dead cells within the biofilms were quantified by BiofilmQ^70^. The number of living and dead cells, the biofilm thickness, and the biofilm density were subjected to a paired sample *t*-test, and the *p*-value was assigned to determine if the two samples were significantly different from each other.

### Flow-based biofilm inhibition test

A flow-cell system (Integrated BioDiagnostics, Martinsried, Germany), as described in^71^, was used for characterization of inhibition effect of Dlh3 on *E. anguillarum* biofilm under flow. An overnight culture of *E. anguillarum* was prepared from a frozen stock in TSB at 30 °C and 200 rpm. Then the culture was centrifuged at 4000 rpm for 10 min, the medium was discarded, and the remaining pellet was resuspended in 5 mL 0.9% NaCl to an OD_600nm_ = 2.82. Three different flow media were prepared from stocks of TSB (20%) and Dlh3 (1.0 mg/mL) as follows; (1) control medium containing 10% TSB diluted in sterile water; (2) test medium adjusted to a final concentration of 10% TSB and 0.5 mg/mL Dlh3; (3) test medium adjusted to a final concentration of 10% TSB and 0.25 mg/mL Dlh3. 14 mL of each of these flow media were filled into 3 separate fluidic units in the flow system. The *E. anguillarum* culture was then inoculated into each of the fluidic units to a final OD_600nm_ = 0.1. The flow parameters were adjusted to a shear stress of 1.19 dyne/cm^2^ in the flow-cell growth channel. To ensure optimal biofilm establishment, the flow-cells were connected to an EVOS FL Auto Imaging system (LifeTechnologies™), and real time biofilm development was monitored visually. The experiment was conducted in a climate laboratory where the temperature was controlled at 30 °C. After 48 h of flow, the flow-cells were disconnected from the flow system, washed carefully with 0.9% NaCl (200 µL), and stained with LIVE/DEAD™ *Bac*Light™ Bacterial Viability Kit. The flow-cells were then incubated in room temperature for 15 min in darkness, and the biofilms formed in the flow-cells were imaged by fluorescens microscopy. Biofilms developed in the flow-cells with 0.5 mg/mL and 0.25 mg/mL Dlh3 were compared to the control flow-cell as a visual assessment of biofilm inhibition.

### Cytotoxicity assay

CHSE-214 cells (kindly gifted from Espen Rimstad, Norwegian University of Life Sciences, NMBU) were cultivated in L-15 medium supplemented with 10% FBS, 2 mM L-glutamine and 10 U/mL PenStrep. For the cytotoxicity assay, 45,000 CHSE-214 cells per well were seeded in a 96-well black wall, non-coated optical bottom plate. Cells were allowed to settle for 30 min before overnight incubation at 20 °C without CO_2_. The next day, a dilution series of Dlh3 was prepared in PBS. Cells were aspirated and the different dilutions of Dlh3 added. For all samples, a fixed volume of 15 µL Dlh3 or plain PBS was mixed with 85 µL cell medium per well. Cells receiving 100 µL cell medium were used as a control sample. All treatments were performed in triplicates. CHSE-214 cells were then incubated for 48 h at 20 °C without CO_2_. Cytotoxicity was determined by the CellTiter-Glo^®^ luminescent cell viability assay (Promega) following the manufacturer's instructions.

### RNA extraction and sequencing of Edwardsiella

Bacteria from an overnight preculture of *E. anguillarum* were adjusted to OD_600nm_ = 0.05. 165 µL of the prepared bacterial culture were pipetted in each of the inner 24 wells of a 48-well plate (Nunclon delta surface; Thermo Fisher Scientific, Waltham, MA, USA). 165 µL of test liquid (protein solution of 0.5 mg/mL Dlh3, control: PBS) were added to the bacterial culture in the inner 24 wells. Triplicates of each test liquid and plates were cultivated at 28 °C for 48 h. To obtain the bacterial biofilm, all supernatants were discarded and the wells were washed by pipetting up and down with one mL of RNAlater™solution (Thermo Fisher Scientific, Waltham, MA) for every eight wells, leading to final 3 mL for each of the six plates. The resulting biofilm suspensions were spun down at 5000 rpm for 30 min and the supernatants discarded. From the remaining cell pellets, total RNA was isolated using a bead mill and the mirVana RNA isolation kit (Ambion) including DNase treatment and library preparation. The six cDNA pools were sequenced on an Illumina NextSeq 500 system. On average 9.7 million (minimum: 9.53 M) single-end sequence reads of length 75 bp were obtained per each sample (vertis Biotechnologie AG, Freising-Weihenstephan, Germany).

### Processing and analysis of RNA-seq reads

Sequence reads were processed with fastp (v0.23.2,^72^) to remove sequences originating from sequencing adapters and sequences of low quality (Phred quality score below 15) from the 3′ end of the sequence reads [FASTP]. The Burrows Wheeler Aligner (BWA v0.7.17,^73^) was used to align the reads to the reference assembly of *E. tarda* EIB202 (ASM2086v1). Counts of reads per gene were obtained using featureCounts (FQ v2.0.3^74^). Differential expression was assessed with DESeq2 (v1.34.0^75^). A gene was considered significantly differentially expressed if the corresponding absolute log2-transformed fold change (log2FC) was not less than 0.5 and, in addition, the false discovery rate (FDR) did not exceed a value of 0.1. The Pathosystems Resource Integration Center (PATRIC,^41^) was used for circular mapping using the integrated Circular Genome Viewer tool. The volcano plot of the distribution of all DEGs was generated using OriginPro V2021b (OriginLab Corporation, USA, VolcanoPlot.opx, 2016).

### Supplementary Information

Supplementary Video 1.

Supplementary Video 2.

Supplementary Information 1.

Supplementary Information 2.

Supplementary Information 3.

Supplementary Information 4.

Supplementary Information 5.

Supplementary Information 6.

Supplementary Information 7.

Supplementary Information 8.

Supplementary Information 9.

Supplementary Information 10.

## Data Availability

Sequence data have been submitted to the European Nucleotide Archive. They are publicly available under accession no. PRJEB61729.
