# Supplementary figures and images for: New dienelactone hydrolase from microalgae bacterial community-Antibiofilm activity against fish pathogens and potential applications for aquaculture
Source: Sci Rep. 2024 Jan 3;14:377. doi: 10.1038/s41598-023-50734-9 (PMC10764354; doi:10.1038/s41598-023-50734-9)

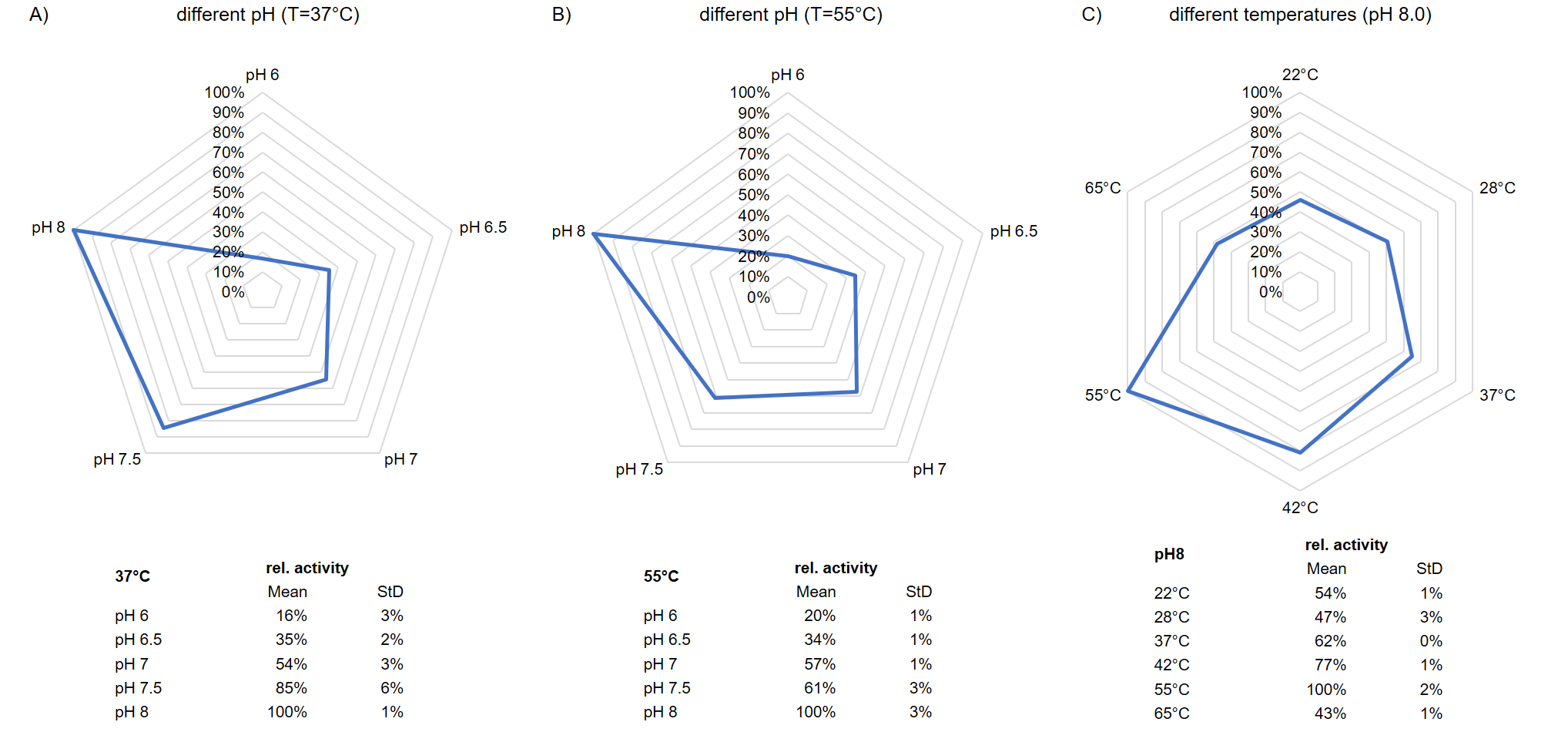

Supplement: Supplementary file 3 — Supplementary Information 1. [file 41598_2023_50734_MOESM3_ESM.tif]

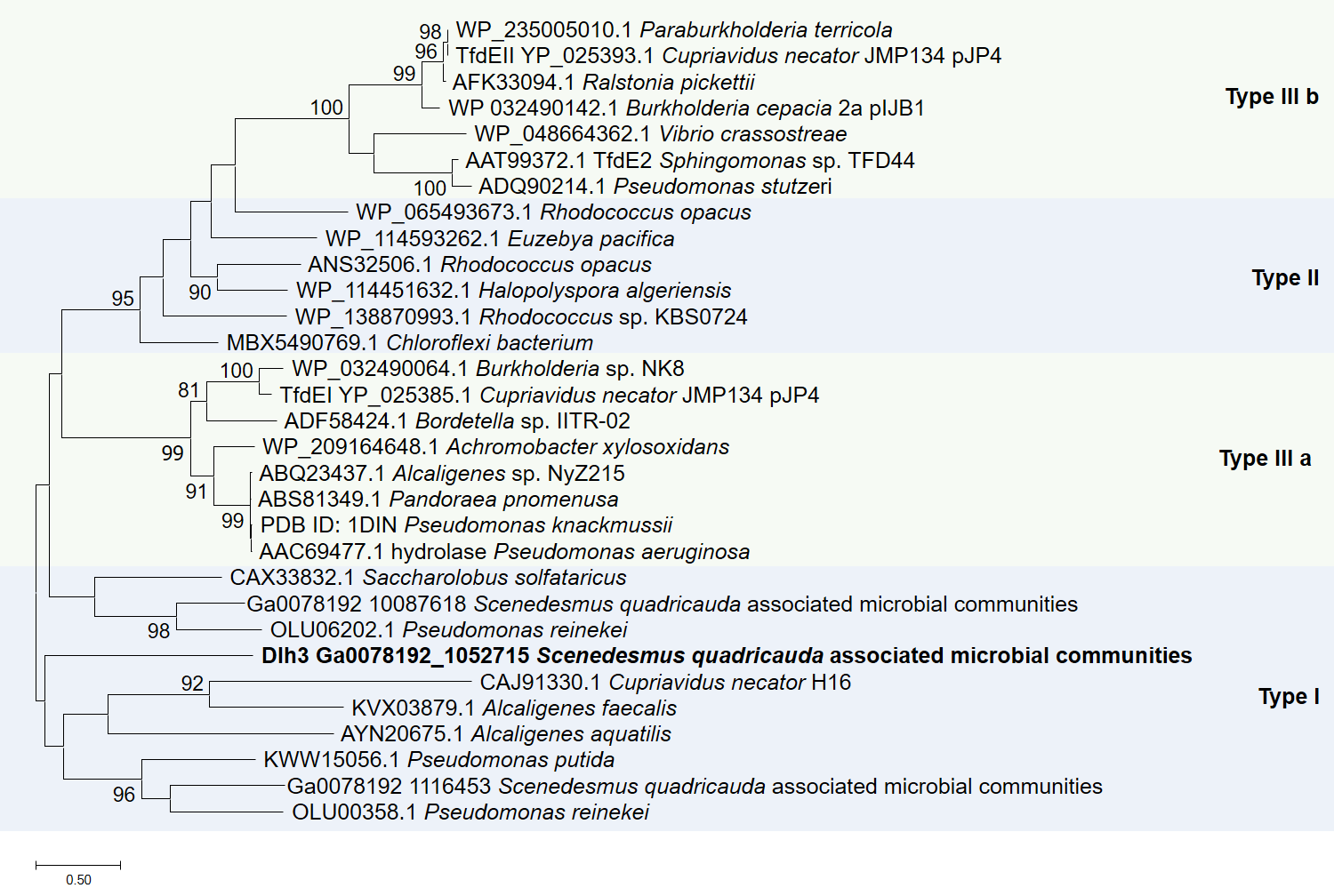

Supplement: Supplementary file 4 — Supplementary Information 2. [file 41598_2023_50734_MOESM4_ESM.tif]

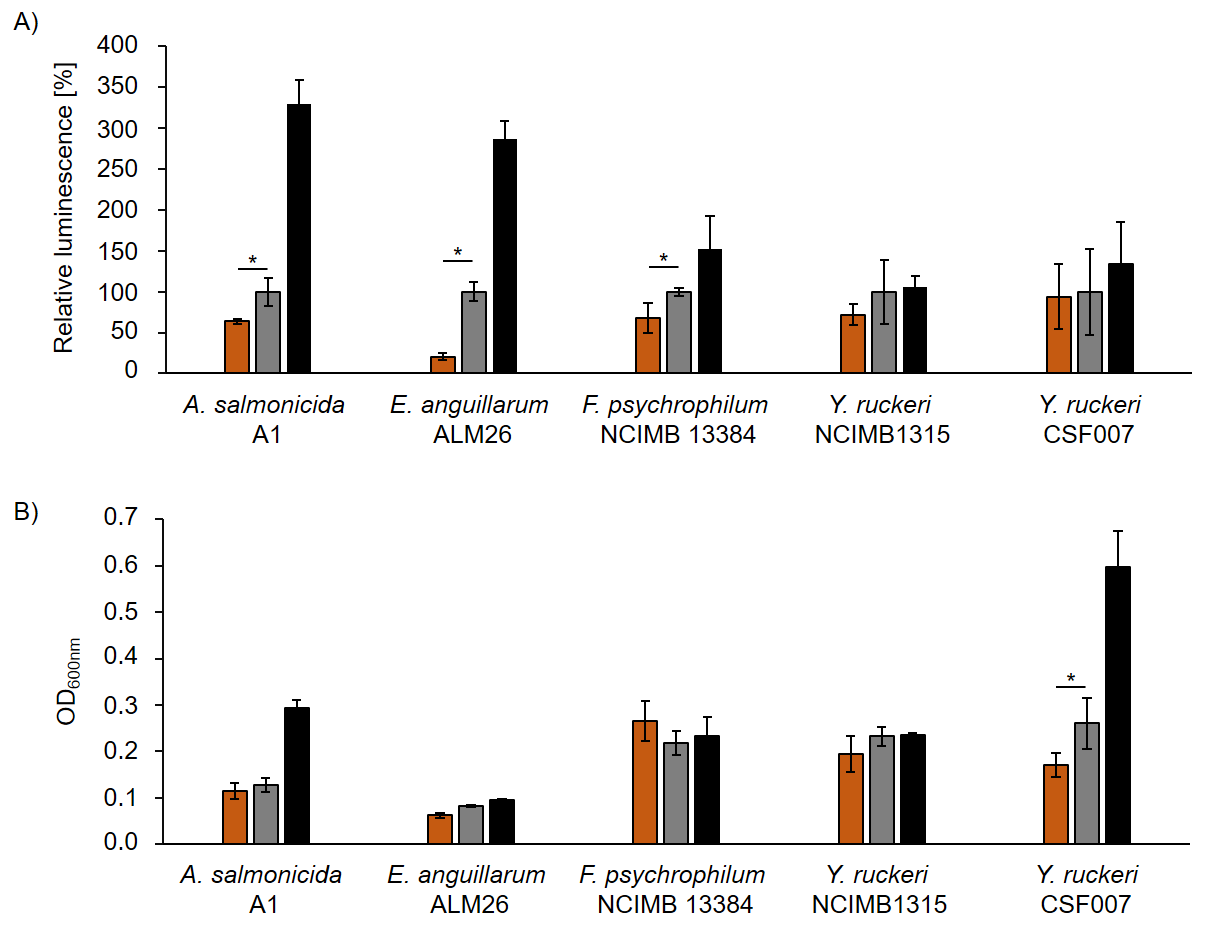

Supplement: Supplementary file 5 — Supplementary Information 3. [file 41598_2023_50734_MOESM5_ESM.tif]

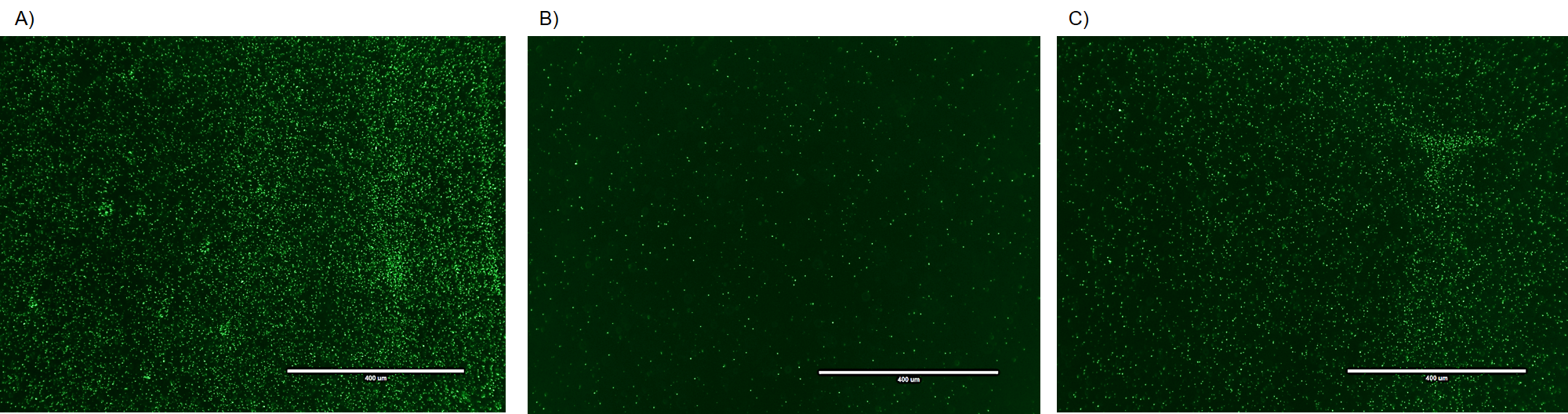

Supplement: Supplementary file 6 — Supplementary Information 4. [file 41598_2023_50734_MOESM6_ESM.tif]

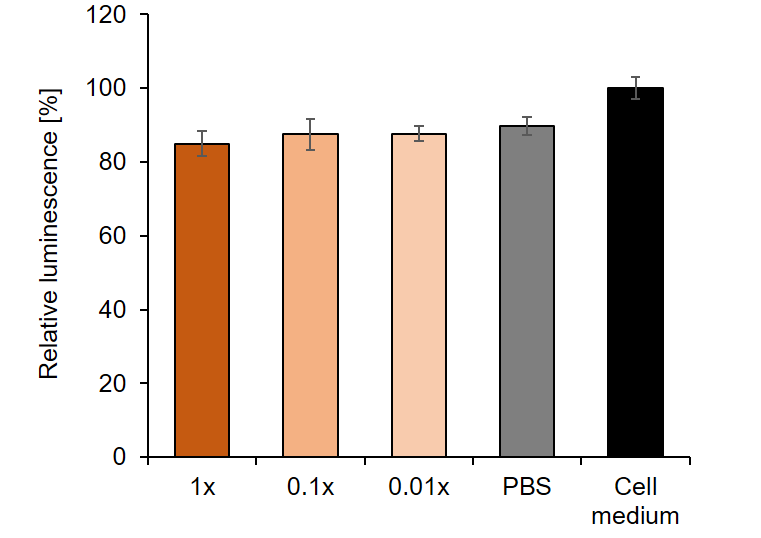

Supplement: Supplementary file 7 — Supplementary Information 5. [file 41598_2023_50734_MOESM7_ESM.tif]
