# Supplementary material for: New dienelactone hydrolase from microalgae bacterial community-Antibiofilm activity against fish pathogens and potential applications for aquaculture
Source: Sci Rep. 2024 Jan 3;14:377. doi: 10.1038/s41598-023-50734-9 (PMC10764354; doi:10.1038/s41598-023-50734-9)
Supplement: Supplementary file 8 — Supplementary Information 6. [file 41598_2023_50734_MOESM8_ESM.docx]

**Table S1:** Key features of potential antibiofilm / antimicrobial agents as well as quorum quenching candidates of microalgae metagenomes communities e.g., *Scenedesmus communis (quadricauda),* *Chlorella saccharophila* and *Micrasterias crux-melitensis* using IMG function search, gene count (date: 12.04.2023). Data shown in total number of hits per 50 Mb.

|  | ***Scenedesmus communis (quadricauda)* community metagenome**  **(IMG ID: 3300005759)** | ***Chlorella***  ***saccharophila* community**  **metagenome**  **(IMG ID: 3300008885)** | ***Micrasterias***  ***crux-melitensis* community**  **metagenome**  **(IMG ID: 3300008886)** |
| --- | --- | --- | --- |
| **Antibiofilm/antimicrobial agents** |  |  |  |
| Polyketide synthases | 43 | 22 | 44 |
| Restriction endonucleases | 11 | 15 | 39 |
| Amylases | 6 | 17 | 30 |
| Chelatases | 27 | 64 | 81 |
| Deaminases | 62 | 206 | 231 |
| Decarboxylases | 80 | 181 | 256 |
| Proteases | 261 | 246 | 329 |
| Lyases | 193 | 293 | 481 |
|  |  |  |  |
| **Quorum quenching agents** |  |  |  |
| Acylases | 6 | 16 | 21 |
| Lactonases | 17 | 66 | 86 |
| Oxidoreductases | 294 | 684 | 866 |
| Hydrolases | 306 | 14 | 24 |
| Dienelactone hydrolase | 15 | 404 | 569 |
|  |  |  |  |
