# Supplementary material for: New dienelactone hydrolase from microalgae bacterial community-Antibiofilm activity against fish pathogens and potential applications for aquaculture
Source: Sci Rep. 2024 Jan 3;14:377. doi: 10.1038/s41598-023-50734-9 (PMC10764354; doi:10.1038/s41598-023-50734-9)
Supplement: Supplementary file 11 — Supplementary Information 9. [file 41598_2023_50734_MOESM11_ESM.docx]

**Table S4:** Bacterial and microalgal strains, plasmids and constructs used in this study

| Strain | Characteristics | Culturing |
| --- | --- | --- |
| *Scenedesmus communis* MZCH10104 | Microalgae community, previously named as *Scenedesmus quadricauda* MZCH10104 | KC, 22°C 4% of CO_2_ at a natural light intensity |
| *Pseudomonas aeruginosa* strain PA14 | gram-negative, aerobic–facultatively anaerobic, rod-shaped, monoflagellated, ampicillin resistance | LB, 37 °C overnight |
| *Edwardsiella anguillarum* strain ALM26 | gram-negative, facultatively anaerobic, rod-shaped, flagellated, chloramphenicol resistance | TSB, 28 °C overnight |
| *Aeromonas salmonicida* strain A1 | gram-negative, facultatively anaerobic, nonmotile, rod-shaped, polymyxin resistance | NB, 28 °C overnight |
| *Flavobacterium columnare* B185R | gram-negative, aerobic, gliding motility, rod-shaped | TYES, 22 °C 72 h |
| *Flavobacterium psychrophilum* NCIMB 13384 | gram-negative, aerobic, gliding motility, rod-shaped | TYES, 22 °C 72 h |
| *Yersinia ruckeri NCIMB1315* | Gram-negative, rod-shaped, facultatively anaerobic, motile | LB, 28 °C overnight |
| *Yersinia ruckeri CSF007* | Gram-negative, rod-shaped, facultatively anaerobic, motile | LB, 28 °C overnight |
| *Escherichia coli* Rosetta-gami^TM^ 2 (DE3) | Δ(*ara-leu*)*7697* Δ*lacX74* Δ*pho A* *Pvu*II *phoR* *araD139* *ahpC galE galK rpsL*(DE3) F′[*lac^+^ lacI^q^ pro*] *gor522*::Tn10 *trxB* pRARE2 (Cam^R^, Str^R^, Tet^R^) | LB, 37 °C overnight |
| pET21a(+) | Vector for expressing His-tagged protein | Novagen/Merck (Darmstadt, Germany) |
| pET21a+::*dlh3* | Expression vector carrying Dlh3 coding gene | This study |
