# Supplementary material for: New dienelactone hydrolase from microalgae bacterial community-Antibiofilm activity against fish pathogens and potential applications for aquaculture
Source: Sci Rep. 2024 Jan 3;14:377. doi: 10.1038/s41598-023-50734-9 (PMC10764354; doi:10.1038/s41598-023-50734-9)
Supplement: Supplementary file 12 — Supplementary Information 10. [file 41598_2023_50734_MOESM12_ESM.docx]

**New dienelactone hydrolase from microalgae bacterial community-Antibiofilm activity against fish pathogens and potential applications for aquaculture**

Lutgardis Bergmann^1^, Simone Balzer Le^2^, Gunhild Hageskal^2^, Lena Preuss^1^, Yuchen Han^1^, Yekaterina Astafyeva^1^, Simon Loevenich^2^, Sarah Emmann^3^, Pablo Perez-Garcia^3^, Daniela Indenbirken^5^, Elena Katzowitsch^6^, Fritz Thümmler^6^, Malik Alawi^4^, Alexander Wentzel^2^, Wolfgang R. Streit^1^ and Ines Krohn^1^*****

^1^ University of Hamburg, Institute of Plant Science and Microbiology, Department of Microbiology and Biotechnology, Hamburg, Germany

^2^ SINTEF Industry, Department of Biotechnology and Nanomedicine, Trondheim, Norway

^3^ Kiel University, Institute for General Microbiology, Molecular Microbiology, Kiel, Germany

^4^ University Medical Center Hamburg-Eppendorf, Bioinformatics Core, Hamburg, Germany

^5^ Leibniz Institute of Virology, Virus Genomics, Hamburg, Germany

^6^ University of Würzburg, Core Unit Systems Medicine, Würzburg, Germany

**Keywords:** healthy aquaculture, fish pathogens, microalgae-bacteria communities, metagenomics, metatranscriptomics, anti-biofilm formation, hydrolase, dienelactone hydrolase

***For Correspondence**

E-Mail: ines.krohn@uni-hamburg.de

Department of Microbiology and Biotechnology,

Institute of Plant science and Microbiology, University of Hamburg,

Ohnhorststr.18

D-22609 Hamburg, Germany,

Tel. (+49) 40-42816-463 /-444

Fax. (+49) 40-42816-459

**Supplementary figure and supplementary table legends**

**Supplemental Figure S1:** **Dlh3 activity assay.** The hydrolase activity of Dlh3 was analyzed with 4-Nitrophenyl octanoate (*p*NP-C8) as substrate. The colorless substrate releases chromogenic para-nitrophenol (yellow) when the ester bond is hydrolyzed, which can then be detected photometric at a wavelength of 405 nm. 0.825 mg/mL of final 5 µL Dlh3 was added to each assay. Each reaction was performed with 2 mM *p*NP-C8 as substrate in 100 mM potassium phosphate buffer with different pH. The reactions were incubated for 1 hour and stopped by adding Na_2_CO_3_ with final concentration of 200 mM. Dlh3 activity at (A) 37°C and (B) 55°C in a buffer with different pH and Dlh3 activity in a buffer with (C) pH 8.0 at different temperatures. Each experiment were measured in triplicates.

**Supplemental Figure S2:** **Phylogenetic tree of different types of dienelactone hydrolases (Dlhs).** Phylogenetic relationships of Dlhs sequences. The sequences of Dlhs were compared with the non-redundant protein database of NCBI by using BLASTP. The phylogenetic tree was constructed with MEGA X [59] based on the Maximum-likelihood method and JTT matrix-based model [60] with 1000 bootstrap replications after multiple alignments with T-Coffee [61]. The percentage of bootstrap resamplings ≥70 is indicated on the branches. The scale bar represents the expected number of changes per amino acids position. The types of Dlhs are classified according to [37]. Type I Dlhs possess trans-dienelactone hydrolysing activity and type II Dlhs only cis-dienelactone, while type III Dlhs are active against both cis- and trans-dienelactones. The phylogenetic tree implies that Type III Dlhs are clustered into two sub-groups, i.e., group a Dlhs exhibit higher catalytic rate to trans-dienelactone, such as Dlh from *Pseudomonas knackmussii* (previous as *Pseudomonus* sp. B13, PDB ID: 1DIN, [33] [37]) and TfDEI from *Cupriavidus necator* JMP134 [38], while group b Dlhs show higher rate of cis-dienelactone conversion, e.g., TfDEII from *Cupriavidus necator* JMP134 [38]. Our phylogenetic tree also shows that type III DLHs from group b have closer evolutional relationship to Type II Dlhs and group a Dlhs are closed to type I Dlhs. Dlh3 found from *Scenedesmus communis* associated microbial communities (this study) is phylogenetically clustered within type I Dlhs, indicating that it is only active on trans-dienelactone.

**Supplemental Figure S3: Static biofilm test BacTiter Glo**™. A) Antibiofilm tests of different fish pathogenic bacterial strains quantified using BacTiter Glo™. Strains were cultivated under static conditions at either 28°C for one day (*E. anguillarum* ALM26), 28°C for two days (*Y. ruckeri* NCIMB1315 and CSF007, and *A. salmonicida* A1) or 15°C for three days (*F. psychrophilum* NCIMB1315 and CSF007). Protein: dienelactone hydrolase (Dlh3), overexpressed in *E. coli* Rosetta-gami^TM^ 2 (DE3), 1x = 0.4 mg/mL. color key: brownish for 1x = 0.4 mg/mL, grey: PBS control, black: bacteria media control. Significant biofilm reduction marked by stars (significance level *p*-value ≤ 0.05). B) Measurement of optical density (OD_600nm_).

**Supplemental Figure S4:** **Flow-based biofilm inhibition test with *Edwardsiella anguillarum* ALM26.** Image micrographs (10X magnification) of (A) control: *E. anguillarum* in 10% TSB. (B) *E. anguillarum* in 10% TSB and 0.5 mg/mL Dlh3. (C) *E. anguillarum* in 10% TSB and 0.25 mg/mL Dlh3. Biofilms were formed under flow conditions at 30°C for 48 h in 10% TSB medium and treated with supplement of Dlh3. The green living biofilm cells attached to the flow-cell are visualized by LIVE/DEAD™ BacLight™ Bacterial Viability staining using fluorescence microscope (EVOS FL Auto Imaging System). No dead cells (red) were detected, the flow of medium will probably detach dead cells from the biofilm.

**Supplemental Figure S5:** **Cell toxicity assay.** Effect of different concentrations of Dlh3 on the growth of fish cell line CHSE-214. On day 1, 45,000 cells in 100 µL medium were transferred to each well of a black, non-coated 96-well plate with optical bottom.

The next day, cells were aspirated and the different concentrations of Dlh3 added. For all samples, a fixed volume of 15 µL Dlh3 (in PBS) or plain PBS was mixed with 85 µL cell medium per well to achieve final Dlh3 of 0.5 mg/mL (1x brownish), 0.05 mg/mL (0.1x orange), 0.005 mg/mL (0.01x beige coloured) or 0 mg/mL (only PBS, grey). Cells receiving 100 µL cell medium were used as a control sample (black). After two days of exposure, the CellTiter-Glo® Luminescent Cell Viability Assay was performed according to the manufacturer's instructions and the relative luminescence (survival) was correlated to the control wells containing pure cell medium. Data represent averages and standard deviations of two biological replicates.

**Supplemental video as part of Figure 2: Predicted Dlh3 structure**

**B**) Movie of active site of Dlh3. Three critical amino acids (C176, D231 and H262 on Dlh3) at the active site are highlighted.

**Supplemental video as part of Figure 2: Predicted Dlh3 structure**

**C)** Movie of comparison of predicted Dlh3 structure (gold) with dienelactone hydrolase from *Pseudomonas* sp. B13 (sky blue, PDB ID: 1DIN, [33]).

**Supplemental Table S1: Key features** of potential antibiofilm / antimicrobial agents as well as quorum quenching agents of metagenome of *Scenedesmus communis* (*quadricauda*), *Chlorella saccharophila* and *Micrasterias crux-melitensis* using IMG function search, gene count. Data shown in total number of hits per 50 Mb.

**Supplemental Table S2: Profile hidden Markov model (HMM**) search for Dlhs (PF01738.21) against the *Scenedesmus communis* metagenome. Each hit (e‑value < E-10) appears sorted by e-value. Taxonomic affiliation was inferred from the best BLASTp hit in the non-redundant protein database of NCBI.

**Supplemental Table S3: Expression analyses.** transcriptome dataset of *Edwardsiella anguillarum* cultured in the presence of with 0.5 mg/mL Dlh3.

**Supplemental Table S4:** **Bacterial and microalgal strains, plasmids and constructs used in this study.**
